# DNA methylation modification is associated with gonadal differentiation in *Monopterus albus*

**DOI:** 10.1186/s13578-020-00490-4

**Published:** 2020-11-10

**Authors:** Xin Wang, Fengling Lai, Jun Xiong, Wang Zhu, Bifeng Yuan, Hanhua Cheng, Rongjia Zhou

**Affiliations:** 1 Hubei Key Laboratory of Cell Homeostasis, College of Life Sciences, Wuhan University, Wuhan, 430072 China; 2 Key Laboratory of Analytical Chemistry for Biology and Medicine of Ministry of Education, Department of Chemistry, Wuhan University, Wuhan, 430072 China

**Keywords:** DNA methylation, Gonad, Promoter, Vertebrates

## Abstract

**Background:**

Both testis and ovary can be produced sequentially in an individual with the same genome when sex reversal occurs in the teleost *Monopterus albus*, and epigenetic modification is supposed to be involved in gonadal differentiation. However, DNA methylation regulation mechanism underlying the gonadal differentiation remains unclear.

**Results:**

Here, we used liquid chromatography-electrospray ionization tandem mass spectrometry (LC–ESI–MS/MS) to simultaneously determine endogenous levels of both 5-methyl-2′-deoxycytidine (m^5^dC) and 5-hydroxymethyl-2′-deoxycytidine (hm^5^dC) during gonadal differentiation. Overall DNA methylation level was upregulated from ovary to testis via ovotestis. As a de novo methylase, *dnmt3aa* expression was also upregulated in the process. Notably, we determined transcription factor Foxa1 for *dnmt3aa* gene expression. Site-specific mutations and chromatin immunoprecipitation showed that Foxa1 can bind to and activate the *dnmt3aa* promoter. Furthermore, DNA methylation levels of key genes *foxl2* (forkhead box L2) and *cyp19a1a* (cytochrome P450, family 19, subfamily A, polypeptide 1a) in regulation of female hormone synthesis were consistently upregulated during gonadal differentiation.

**Conclusions:**

These data suggested that dynamic change of DNA methylation modification is associated with gonadal differentiation.

## Introduction

DNA cytosine methylation (DNA methylation) is a major epigenetic modification that plays vital roles in animal development. During germ cell development, epigenetic reprogramming occurred dynamically, remodeling of DNA methylation marks in particular [1]. At day 7.5 of early embryo, global DNA methylation of primordial germ cells (PGCs) were erased and new DNA methylation were then established during late stage of development in mouse [2]. However, de novo DNA methylation proceeded differentially between male and female germ cells, earlier in spermatogenesis than in oogenesis.

In the female germline cells, de novo DNA methylation occurred in arrested oocytes in meiotic prophase I. However, in the male germline cells, it took place in mitotically arrested prospermatogonia before birth [3, 4]. Reprogramming of DNA methylation not only occurs during germ cell differentiation, but also after fertilization. DNA methylation marks in sperms were quickly erased in the zygote by active demethylation mechanism [5], in contrast, the methylation marks in oocytes were lost via passive dilution through cell division [1].

The DNA methylation was established by DNA methyltransferases, the DNA methyltransferase (cytosine-5) (DNMT) family proteins, including Dnmt1, Dnmt3a, Dnmt3b, and Dnmt3l, while active demethylation was performed by Ten-eleven translocation (TET) family proteins, consisting of Tet1, Tet2, and Tet3. In DNMT family, Dnmt1 maintained DNA methylation patterns and it worked after DNA replication, and Dnmt3a and Dnmt3b were de novo methylases [6]. Dnmt3l was a regulatory factor for Dnmt3a and Dnmt3b, owing to an enzymatically inactive form of DNMT [7]. Both Dnmt3a and Dnmt3l, but not Dnmt3b, were required for gametogenesis. In mice, methylation in oocytes with KO Dnmt3a or Dnmt3l was not acquired during oocyte growth, leading to abnormal embryonic development [7, 8]. In the *Dnmt3a*^−/−^ testis, meiotic catastrophe was observed in germ cells, and the number of spermatocytes was greatly reduced, owing to impaired chromosome synapsis [9, 10]. Tet proteins, as 5-methylcytosine (5mC) dioxygenases, can converse 5mC to be 5-hydroxymethylcytosine (5hmC), 5-formylcytosine (5fC), and 5-carboxylcytosine (5caC) [11]. These oxidized forms of 5mC would be excised by thymine DNA glycosylase (TDG), together with base excision repair (BER), and the Tet-dependent action resulted in active DNA demethylation [12–14].

The forkhead box (FOX) family consisted of over 100 proteins with subfamilies ranging from FOXA to FOXS, and mutations of them were associated with human diseases including cancers [15]. As transcription factors, FOX proteins contained a conserved DNA binding domain with ~ 100 amino acids [16]. Binding site of Fox proteins contained a core sequence [T(A/G)TT(T/G)], but different subfamilies can recognize diverse adjacent sequences [17]. Foxa1, belonging to Foxa subfamily, was pioneer factor participating in epigenetic switch. Foxa1 often bound to the genomic regions with local DNA hypomethylation [18]. The demethylation was involved in Tet proteins. Foxa1 was associated with Tet1 cis-regulatory elements and induced its expression, which occurred via direct interaction of Foxa1 with the Tet1 protein to co-occupy enhancers and mediate local DNA demethylation [19].

Gonadal differentiation of the teleost *Monopterus albus* occurred naturally from ovary to testis via an intermediate stage, ovotestis [20]. Both male and female possess the same genome, thus epigenetic modification of the genome is supposed to be one of important mechanisms for gonadal differentiation. However, epigenetic regulation mechanisms underlying the gonadal differentiation remains unclear. In this report, we reported DNA methylation levels of three stages of gonadal differentiation by sensitive LC–ESI–MS/MS analysis [21, 22] and simultaneously determined two cytosine modifications, m^5^dC and hm^5^dC. We further characterized transcription factor Foxa1 for *dnmt3a* gene expression, which can activate the *dnmt3aa* promoter. In addition, upregulation of DNA methylation levels in *foxl2* and *cyp19a1a* promoters was associated with gonadal differentiation.

## Materials and methods

### Experimental animals

The fish *Monopterus albus* individuals were obtained from local markets in Wuhan, Hubei Province, China. All animal experiments and methods were performed in accordance with the relevant approved guidelines and regulations, as well as under the approval of the Ethics Committee of Wuhan University.

### Gonad preparation

Gonads were collected and washed twice in 1xPBS. The tissues were cut into a series of 6 µm sections using a cryostat (Leica, Bensheim, Germany). Sex was verified by histological analysis of the gonad sections. The sections were stained by haematoxylin and eosin and images were captured using a Leica microsystem (Leica).

### RNA isolation and RT-PCR

Total RNAs of gonad tissues were isolated using the TRIzolReagent (15596-026, Thermo Fisher) and then were treated with RNase-free DNase (M610A, Promega, Madison, WI, USA). The total RNAs were reverse-transcribed to complementary DNAs (cDNAs) using MMLV (M1701, Promega). PCR was performed using the cDNA as a template and the primers listed in Additional file 1: Table S1.

### Plasmid constructs

Full-length *foxa1* (NW_018128138) was cloned into pcDNA3.0 using EcoRI and XhoI to generate pCMV-*foxa1*. Four deletion fragments of the *dnmt3aa* (NW_018127943) promoter were amplified from genomic DNA, and cloned into pGL3-basic vector (E1751, Promega, Madison, WI, USA) using KpnI and XhoI. The primers and PCR conditions were described in Additional file 1: Table S1. Site-directed mutagenesis for the Foxa1 binding sites were performed using the primers described in Additional file 1: Table S1. All constructs were sequenced.

### Chromatin immunoprecipitation (ChIP)

Chromatin immunoprecipitation was performed according to our previous study [23]. Briefly, testis samples were minced with sterilized scissors in PBS and a final concentration of 1% formaldehyde-PBS was used for crosslinking for 20 min at room temperature. Glycine was added to terminate crosslinking in a final concentration of 0.125 mol/l. The supernatant chromatin was immunoprecipitated with anti-Foxa1 (GTX100308, GeneTex, Alton Pkwy Irvine, USA), no antibody (beads only), or preimmune IgG, together with protein G PLUS-agarose (Sc-2002, Santa Cruz, USA). The DNA isolated from the immunoprecipitated complex was amplified by PCR using relevant primers (Additional file 1: Table S1). The PCR products were cloned into T-easy vector (A137A, Promega, Madison, USA) and sequenced.

### Cell culture, transfection and dual-luciferase reporter assays

HEK293T (3142C0001000001715) and COS7 (3142C0001000000088) cells were obtained from China Center for Type Culture Collection and cultured in high glucose Dulbecco’s modified Eagle’s medium (DMEM) (SH30022.01B, HyClone, Logan, USA) with 10% fetal bovine serum (P30-330250, PAN-Biotech, Aidenbach, Germany) in 6/48-well plates and LipofectamineTM 2000 (11668027, Invitrogen, CarIsbad, USA) was used for transfection according to the routine protocol. For luciferase assays, cells per well was transfected with 0.5 μg recombinant constructs and 1 ng pRL-TK (E2241, Promega Madison, USA). Then luciferase activities were measured by a dual-luciferase reporter assay system (Promega) and a Modulus Single Tube Multimode Reader (Turner Biosystems, Sunnyvale, CA, USA) according to the manufacturer’s protocol. The experiments were repeated at least 3 times, and the results were expressed as the mean ± SEM.

### Enzyme digestion of genomic DNA

The genomic DNA of gonads were extracted using DNAiso Reagent (Takara Biotechnology, Dalian, China) according to the manufacture’s recommended procedure. The concentration of the genomic DNA was determined by a BioPhotometer (RS232C, Eppendorf, Hamburg, Germany). Genomic DNA was digested by S1 Nuclease (EN0321, Thermo Scientific, Rockford, IL, USA) at 37 °C with water bath for 16 h, and the total volume of reaction system was 10 µl with 10 µg genomic DNA, 1 µl 10×S1 Nuclease buffer, 90 U S1 Nuclease. Then, 9 U alkaline phosphatase (18009027, Invitrogen, Carlsbad, USA) and 0.002 U phosphodiesterase I (P4506, Sigma-Aldrich, St Louis, USA) were added to the reaction system of 40 µl simultaneously. The reaction was incubated in water bath at 37 °C for 4 h. After enzyme digestion, the samples were extracted by phenol and chloroform. The nucleoside samples were analyzed by LC–ESI–MS/MS.

### Determination of m^5^dC and hm^5^dC by LC–ESI–MS/MS analysis

The liquid chromatography-electrospray ionization tandem mass spectrometry (LC–ESI–MS/MS) were performed using the method described previously with slight modification [22, 24]. The LC–ESI–MS/MS system consisted of a Shimadzu LC-20AD high performance liquid chromatography (HPLC) (Tokyo, Japan) and an AB 3200 QTRAP mass spectrometer (Applied Biosystems, Foster City, CA) with an ESI source (Turbo Ionspray). Data were acquired and processed by using AB SCIEX Analyst 1.5 Software (Applied Biosystems, Foster City, CA, USA). The LC separation was performed on a Shimadzu VP-ODS column (250 mm × 2.1 mm i.d., 5 µm, Tokyo, Japan) with a flow rate of 0.2 ml/min at 40 °C. 2 mM NH4HCO3 in water (solvent A) and methanol (solvent B) were employed as mobile phase. A gradient of 0–5 min 5% B, 5–15 min 5–25% B, 15–28 min 25–70% B and 30–40 min 5% B was used. The mass spectrometry detection was performed under positive ESI mode. The nucleosides were monitored by multiple reaction monitoring (MRM) mode. The MRM parameters of all nucleosides were optimized to achieve maximal detection sensitivity. Mass spectrometry parameters for detection of nucleosides were listed in Additional file 1: Table S3. 2′-Deoxycytidine (dC) (D3897) was purchased from Sigma -Aldrich (St. Louis, MO, USA). 5-methyl-2′- deoxycytidine (m^5^dC) (PY7587) and 5-hydroxymethyl-2′-deoxycytidine (hm^5^dC) (PY7588) were purchased from Berry & Associates (Dexter, MI, USA).

### Bisulfite PCR methylation analysis

Bisulfite PCR methylation analysis method was described previously [25]. Briefly, 2 µg of genomic DNAs of ovary, ovotestis, and testis were denatured with 3 M NaOH for 10 min at 37 °C, then treated with 3 M sodium bisulfite (pH 5.0) (RH149641, RHAWN, Shanghai, China) and 10 mM hydroquinone (123K2508, Sigma-Aldrich) at 55 °C for 16 h. Treated genome was purified by purification kit (D2022, US EVERBRIGHT, Suzhou, China). Finally, PCR amplification was performed using the primers for 5′ flanking region of *foxl2* described in Additional file 1: Table S1 and the primers for *cyp19a1a* described in previous study [26]. PCR products were sequenced.

### Bioinformatic analysis

Prediction of transcriptional factor binding sites was performed by JASPAR [27] and PROMO [28, 29]. CpG island analysis was performed by MethPrimer software (http://www.urogene.org/methprimer2).

### Statistical analysis

All data were presented as mean ± standard error. Statistical comparisons were made using Student’s *t* test when comparing two groups. One-way ANOVA was used for comparisons with more than two groups. Statistics analysis was performed by Dunnett’s multiple comparisons and tests were performed using GraphPad Prism (v7.00 for Windows, GraphPad Software, La Jolla California, USA). For determination of m^5^dC and hm^5^dC, over 3 biological repeats were performed for each sex. For DNA methylation of promoters, 10 clones were analyzed for each site. P < 0.05 was considered to be statistically significant.

## Results

### Simultaneous determination of m^5^dC and hm^5^dC in gonadal genome during gonadal differentiation by LC–ESI–MS/MS

**Fig. 1:**
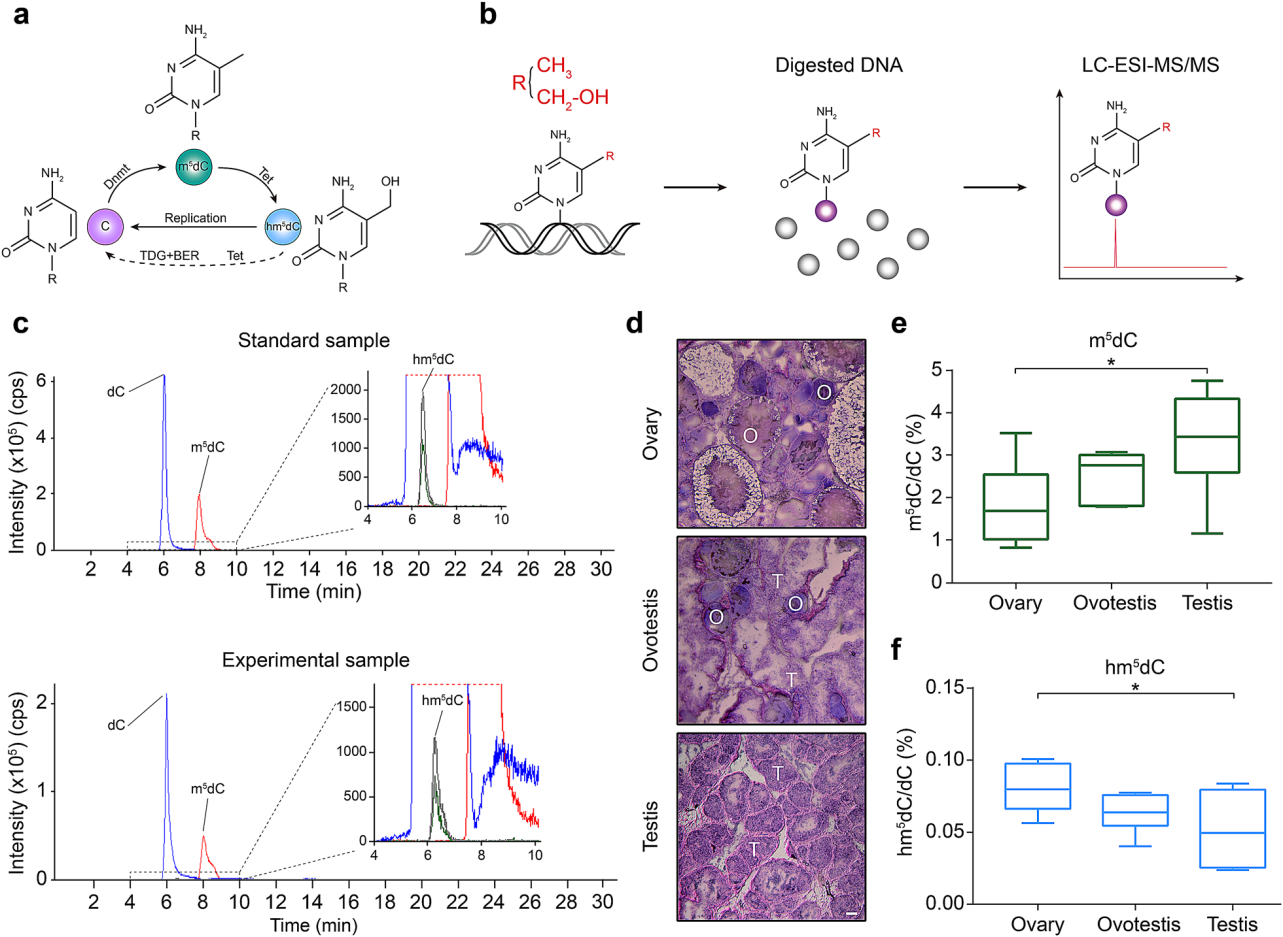
Dynamic change of methylation and hydroxymethylation levels among ovary, ovotestis, and testis. **a** Schematic showing cycle of 5mC and 5hmC converted from and to unmodified cytosine by Dnmts, Tet, and replication-dependent passive demethylation or via 5-formylcytosine (5fC) and 5-carboxylcytosine (5caC). **b** The flow chart of m^5^dC and hm^5^dC analysis using enzyme digestion and LC–ESI–MS/MS. **c** Chromatograms for detection of 2′-Deoxycytidine (dC), m^5^dC, hm^5^dC from standard sample (top panel) and experimental sample (bottom panel). **d** Histology of gonadal tissues (ovary, ovotestis, and testis) stained by hematoxylin and eosin. O, ovary; T, testis. Scale bar, 100 µm. **e** Box plot illustrating the levels (%) of m^5^dC (m^5^dC/dC) among ovary, ovotestis and testis, the mean ± SEM were from more than 3 independent experiments. **f** Box plot showing the levels (%) of hm^5^dC (hm^5^dC/dC) among ovary, ovotestis and testis, the mean ± SEM were from more than 3 independent experiments, *P < 0.05

DNA cytosine methylation, 5mC was performed via methyl addition by Dnmt family proteins. However, the methylation can partly be reversed to 5hmC by Tet family proteins and further to its unmodified state by replication dependent dilution or thymine DNA glycosylase (TDG)/base excision repair (BER) pathways (Fig. 1a). It is often difficult to detect endogenous 5hmC, owing to its low abundance. We used LC–ESI–MS/MS to simultaneously determine endogenous levels of both 5-methyl-2′-deoxycytidine (m^5^dC) and 5-hydroxymethyl-2′-deoxycytidine (hm^5^dC) during gonadal differentiation (Fig. 1b). The extracted-ion chromatograms of both standards and gonadal samples showed the distinct determination of these modifications (Fig. 1c), meanwhile, the coefficient of determination (R^2^) of linearity was higher than 0.99 (Additional file 1: Table S2), indicating high accuracy and precision for the determination. LC–ESI–MS/MS showed that median value (%) of m^5^dC was increased from ovary to ovotestis to testis with significant difference (1.864 vs 3.282) between ovary and testis (P < 0.05) (Fig. 1d, e). In contrast, median value (%) of hm^5^dC was obviously decreased during the gonadal differentiation, and the difference between ovary and testis (0.08 vs 0.0519) was also significant (P < 0.05) (Fig. 1f). Together, these results suggested that DNA methylation level was upregulated during the gonadal differentiation.

### Identification of regulatory elements for *dnmt3aa* expression

**Fig. 2:**
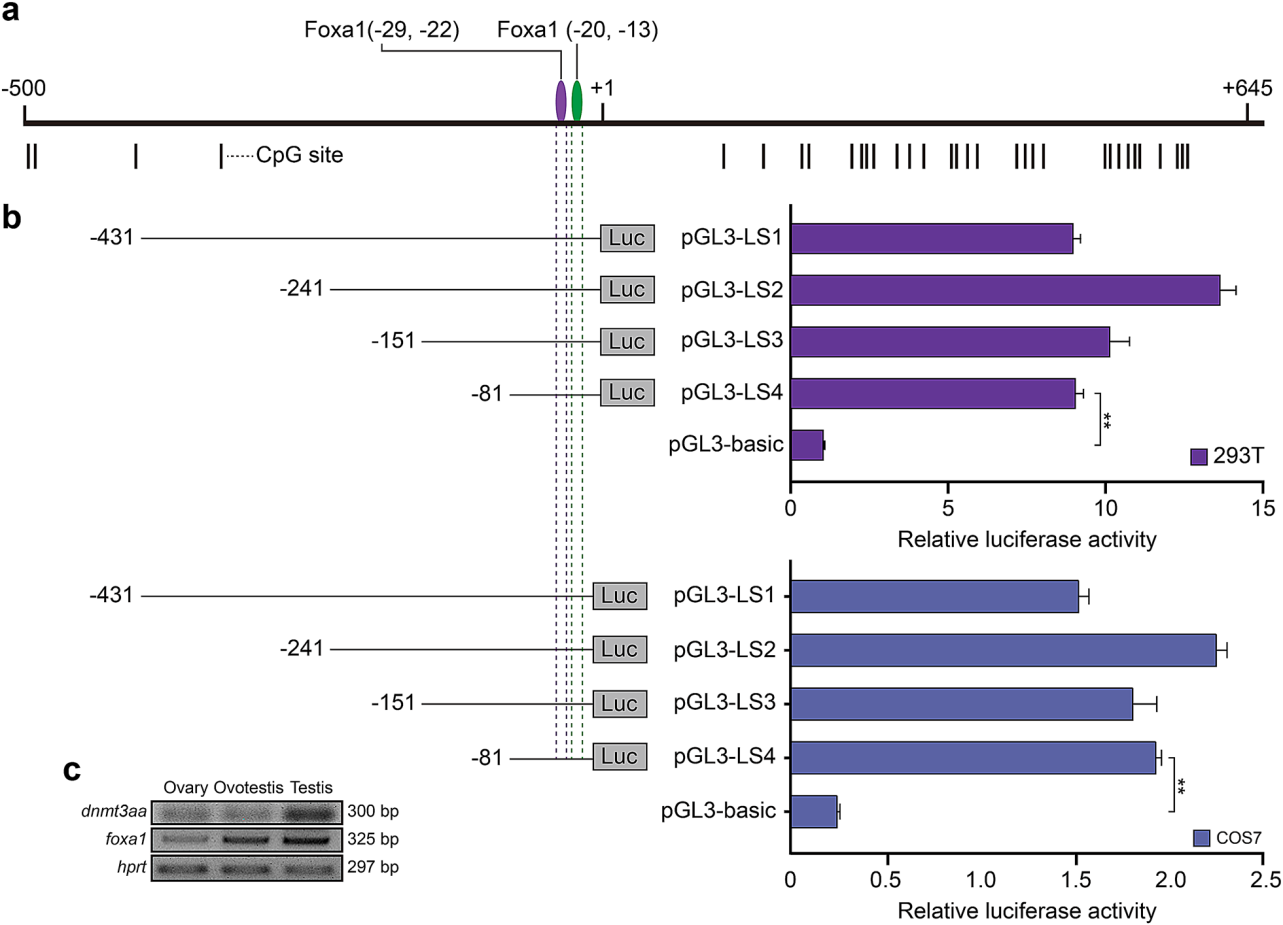
Activity analysis of the *dnmt3aa* promoter. **a** Schematic showing the position of Foxa1 binding sites and CpG sites. Two Foxa1 binding sites, **a** from − 13 to − 20, and **b** from − 22 to − 29; CpG sites were represented by short bars. **b** Luciferase assay showing the activities of a series of deleted constructs in 293T (top panel) and COS7 (bottom panel) cells. Left part of each panel showed each deleted mutant linked with the luciferase gene in the pGL3-basic vector. Right part indicated the relative activities of these deleted constructs. The mean ± SEM were from 3 independent experiments, *P < 0.05; **P < 0.01. **c** Expression pattern of *dnmt3aa* and *foxa1* among gonads analyzed by semi-quantitative RT-PCR. *hprt* (hypoxanthine phosphoribosyltransferase) was employed as an internal control

To explore expression regulation of *dnmt3aa*, we analyzed sequence of promoter region by MethPrimer software. We found that there was no CpG island in the core promoter region, and TATA box was also lacked. Potential binding sites for transcriptional factors were then predicted by JASPAR and PROMO. We found two forkhead box (− 13 bp to − 20 bp; − 22 bp to − 29 bp) in the core promoter region (Fig. 2a). To determine the exact binding sites, luciferase reporter with a series of deletions was used. Luciferase activities of four deletion constructs were high including LS4 (− 1 bp to − 81 bp) (Fig. 2b), and the results of two cell lines (293T and COS7) indicated that key cis elements were indeed in -1 bp to -81 bp, which contained two tandem forkhead boxes. We further performed RT-PCR to analyze expression patterns of *dnmt3aa* and *foxa1* among ovary, ovotestis and testis. The PCR result showed that expressions of both *dnmt3aa* and *foxa1* were upregulated during gonadal differentiation (Fig. 2c), indicating a similar expression trend between *dnmt3aa* and *foxa1*.

### Foxa1 activates the *dnmt3aa* promoter

**Fig. 3:**
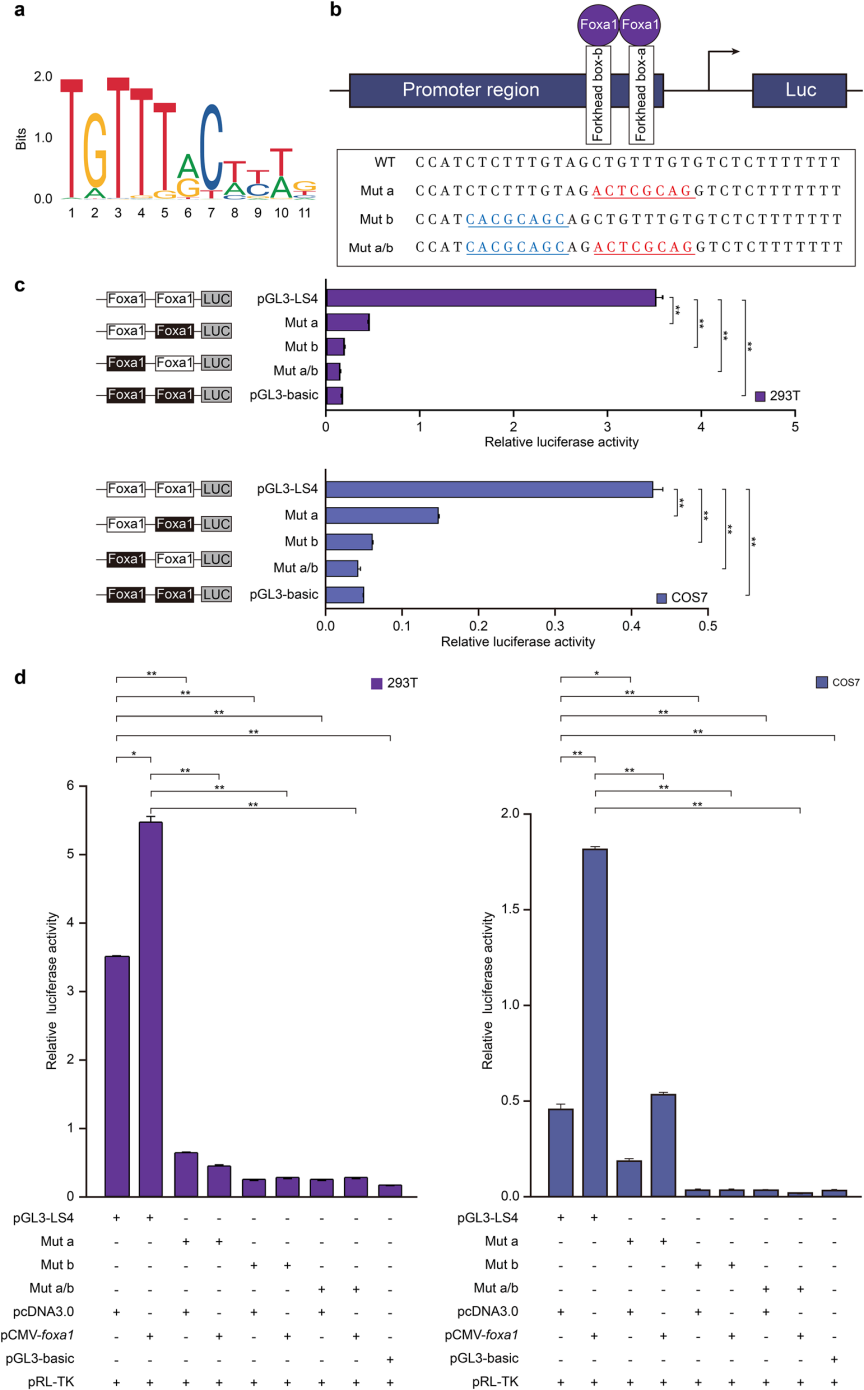
Mutation analysis of Foxa1 binding sites in the *dnmt3aa* promoter. **a** Sequence logo of Foxa1 binding site predicted by JASPAR database (jaspar.genereg.net) with core sequence [T(A/G)TT(T/G)]. **b** Schematic showing the DNA sequences of *dnmt3aa* mutants and wild type. Mut a, b and a/b indicated mutant site a (red), b (blue), and a/b double mutant respectively. **c** Point mutation analysis of the promoter using luciferase assays in 293T (top panel) and COS7 (bottom panel) cells. Both mutations a and b can lead to obviously decrease of luciferase activity, and the decrease was most apparent in double mutant. The mean ± SEM were from 3 independent experiments, *P < 0.05; **P < 0.01. **d** Overexpression of *foxa1* up-regulated *dnmt3aa* promoter activities in 293T (left panel) and COS7 (right panel) cells. The mean ± SEM were from 3 independent experiments, *P < 0.05; **P < 0.01

To determine potential role of the transcription factor Foxa1 in the *dnmt3aa* promoter activity, site-directed mutants were constructed using wild-type pGL3-LS4 plasmid as the template. Three mutants were obtained, including single site mutants a and b, and double site mutant a/b (Fig. 3a, b). Compared with the wild-type pGL3-LS4 construct, single site mutant a or b showed an obvious decrease in luciferase activity, and double mutants revealed a scarce activity in both cell lines (Fig. 3c). Moreover, overexpression of *foxa1* can significantly increase LS4-driven luciferase activity, in contrast, it scarcely activated on the single or double sites mutation-driven luciferase activity (Fig. 3d). The results suggested that Foxa1 could bind foxhead-box to activate the *dnmt3aa* promoter, meanwhile, foxhead-box a and b could not work independently, and mutation of any of them resulted in an obvious decrease of the promoter activity.

### Foxa1 binds to the *dnmt3aa* promoter in vivo

**Fig. 4:**
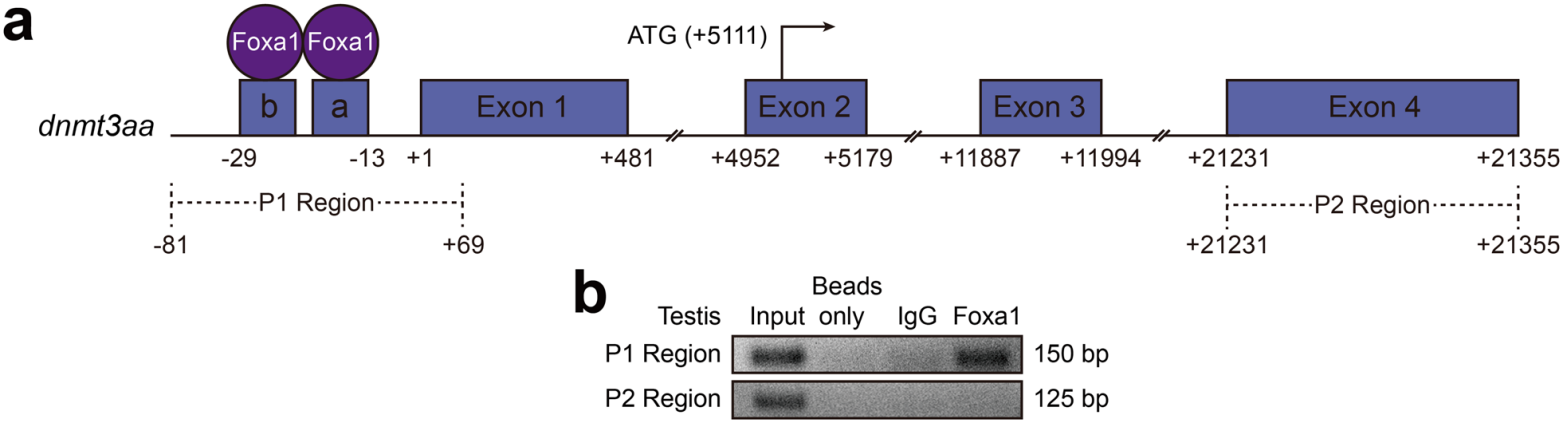
Chromatin immunoprecipitation assays. **a** Schematic illustration showing primer positions of *dnmt3aa* gene in the ChIP assay. P1 region and P2 region were target region and control region, respectively. **b** Chromatin immunoprecipitation in vivo. Foxa1 can bind to the *dnmt3aa* promoter in testis. P2 region was used as a control. Sonicated chromatin from testis were immunoprecipitated with anti-Foxa1, beads only, and preimmune IgG (control). A 150 bp fragment corresponding to the −81 to +69 region was amplified using the immunoprecipitated DNA as a template. PCR amplification of Exon 4 of *dnmt3aa* was used as a negative control

Chromatin immunoprecipitation analysis was further performed to investigate whether Foxa1 binds to the *dnmt3aa* promoter in vivo. A 150 bp DNA region from anti-Foxa1 antibody precipitates was amplified in testis. Another DNA fragment in distinct genomic region (exon 4) was used as a control to exclude the possibility of non-specific binding to the *dnmt3aa* promoter region (Fig. 4a, b). These results suggested that Foxa1 can bind to foxhead box a and b in the *dnmt3aa* promoter and activate the promoter activity in testis.

### DNA methylation of *foxl2* and *cyp19a1a* promoters is associated with gonadal differentiation

**Fig. 5:**
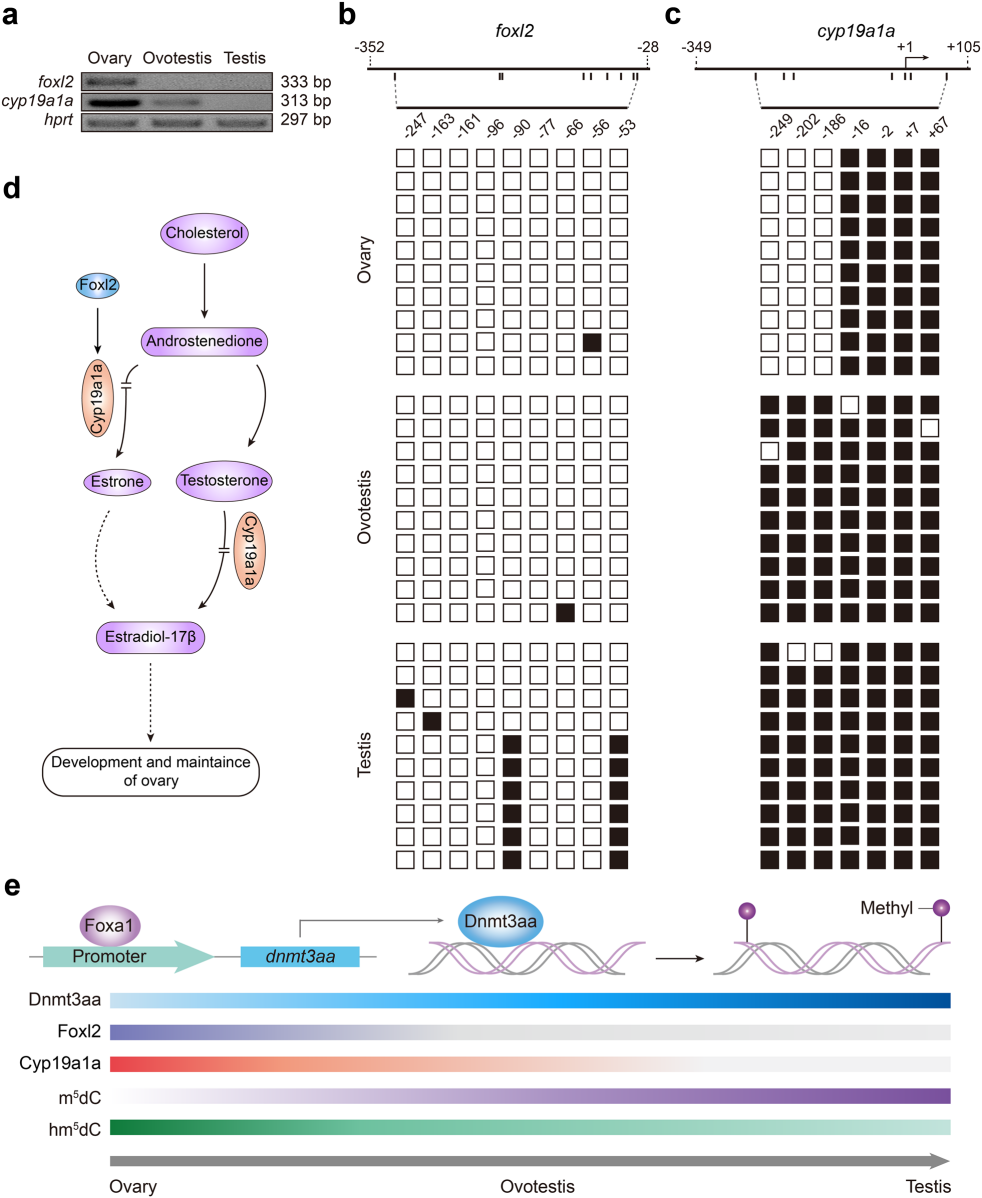
Expression regulation of *foxl2* and *cyp19a1a* by DNA methylation**. a** *foxl2* and *cyp19a1a* expression pattern among gonads by semi-quantitative PCR. *hprt* was employed as an internal control. **b** Methylation status of CpG dinucleotide of *foxl2* 5′ flanking region in ovary, ovotestis, and testis. Open box and black box represented unmethylated site and methylated site respectively. **c** Methylation status of CpG dinucleotide of *cyp19a1a* 5′ flanking region in ovary, ovotestis, and testis. Open box and black box represented unmethylated site and methylated site respectively. **d** Schematic depiction of synthesis pathway of female hormone estradiol-17β, in which expressions of *foxl2* and *cyp19a1a* were associated with DNA methylation status in their promoters. **e** A model showing DNA methylation regulation in gonadal differentiation. A balance mechanism between upregulation of DNA methylation m^5^dC and downregulation of hydroxymethylation hm^5^dC plays an important role in gonadal differentiation. Foxa1 binds to *dnmt3aa* promoter and activates its expression in gonads. DNA methylation levels are downregulated in promoters of key genes *foxl2* and *cyp19a1a* in female determination pathway, which enhances their expression, thus promotes development and function maintenance of ovary, while in testis, Dnmt3aa expression causes DNA methylation of *foxl2* and *cyp19a1a* promoters, and decreases their expression, thus suppresses female determination pathway

Foxa1 can activate *dnmt3aa* transcription, which probably upregulates the methylation levels of key genes in gonad differentiation, including *foxl2* and *cyp19a1a*. The Foxl2-Cyp19a1a pathway was essential for development and function maintenance of ovary [23]. To further explore association of DNA methylation with gonad development, we analyzed DNA methylation levels of the key genes in the synthesis pathway of female hormone estradiol-17β. RT-PCR showed that expression levels of *foxl2* and *cyp19a1a* were highest in ovary and decreased in ovotestis, and scarce expression in testis can be detected (Fig. 5a). DNA methylation levels in their promoters were further analyzed. Bisulfite PCR methylation analysis showed that 60% of both − 53 and − 90 CpG sites of 5′ flanking region of *foxl2* were methylated in testis, in contrast, they were hypomethylated in both ovary and ovotestis (Fig. 5b). As a transcriptionally regulated gene by Foxl2 [30], methylation of the *cyp19a1a* promoter was observed in gonads [26]. Indeed, 7 CpG sites were nearly methylated in ovotestis and testis, however, − 249, − 202 , − 186 sites were almost unmethylated in ovary (Fig. 5c). These results indicated the DNA methylation levels of 5′ flanking regions of both *foxl2* and *cyp19a1a* were associated with their expression during gonadal differentiation. Both Foxl2 and Cyp19a1a promoted estrogen synthesis for ovary development [23, 31] (Fig. 5d). Taken together, these data suggested that Foxa1 activated *dnmt3aa* transcription, which upregulated the methylation levels of key genes, for example *foxl2* and *cyp19a1a*, and suppressed the expression of these genes in testis differentiation. Hypomethylation in their promoters facilitated the expression of *foxl2* and *cyp19a1a* genes for ovary development (Fig. 5e). Thus, upregulation of DNA methylation levels of *foxl2* and *cyp19a1a* promoters was associated with gonadal differentiation from ovary to testis.

## Discussion

DNA methylation is a major form of epigenetic modification and has a widespread influence on gene expression in many cellular processes, including germ cell differentiation and maturation [1]. In the present study, we have simultaneously determined endogenous levels of both m^5^dC and hm^5^dC in gonads using sensitive LC–ESI–MS/MS technology, and found upregulation of DNA methylation levels during gonadal differentiation, suggesting a dynamic balance between 5mC and hydroxymethyl 5hmC to maintain an overall methylation level. The hypermethylation is probably important for gonadal differentiation of the teleost *Monopterus albus* from ovary to testis via ovotestis [20]. Consistent with this view, DNA methyltransferase 3 (Dnmt3aa) was upregulated during the differentiation.

How *dnmt3aa* expression is regulated in gonad development remains an open question. As DNA methyltransferases, Dnmt3a and Dnmt3b were responsible de novo DNA methylation [6]. There were multiple members of Dnmt3 in both mammals [32] and teleosts [33]. Two *dnmt3a* members (*dnmt3aa* and *dnmt3ab*) have been detected in the genome of *Monopterus albus* [34], which would be duplicated through whole-genome duplication event [35]. To further explore association of de novo DNA methylation with gonadal differentiation, we characterized transcription regulation of *dnmt3aa* and found that the transcription factor Foxa1 was responsible for transcriptional activation of *dnmt3a* gene. Foxa1 can bind to and activate the *dnmt3aa* promoter both in vitro and in vivo. Foxa1 was proposed as a key factor in epigenetic switch [18, 19]. In addition, Foxa1 can also promote active demethylation via inducing Tet1 expression at transcriptional level and interacting with Tet1 directly [19]. Together, these data suggested that Foxa1 could exerts roles in regulation of DNA methylation through dynamic balance between methylation and demethylation in diverse conditions.

To further explore whether change of DNA methylation levels in particular genes is associated with gonadal differentiation, we determined DNA methylation levels of key genes in the pathway of sex hormone synthesis. The estrogen estradiol-17β was essential for development and function maintenance of ovary [23, 31], and its synthesis was regulated by Foxl2-Cyp19a1a pathway [23, 30]. As expected, DNA methylation levels of the promoters of both *foxl2* and *cyp19a1a* genes were upregulated during gonadal differentiation. Two methylated GpC (− 53 and − 90) in the *foxl2* promoter are potentially important sites for its expression in regulation of the target *cyp19a1a*. Interestingly, two transcription factors Foxc1 and Etv5 were predicted for binding to the − 53 and − 90 GpC sites respectively. Considering the fact that *foxc1* knockout disrupted migration of primordial germ cells and follicles development in mice [36] and Etv5 was also expressed in granulosa cells during folliculogenesis in mice [37], Foxc1 and Etv5 were probably important in regulation of *foxl2* expression through DNA methylation in gonadal differentiation, which remain to be studied further. Three members, *foxl2, foxa1*, and *foxc1* belong to Forkhead-box (FOX) gene family [15]. Foxa1 can activate *dnmt3aa* transcription, which upregulated the methylation levels of key genes in gonad differentiation, including *foxl2* and *cyp19a1a*, and suppressed the expression of these genes in testis differentiation, whereas hypomethylation in their promoters enhanced transcription of both *foxl2* and *cyp19a1a* genes for ovary development. Thus, the members, *foxl2, foxa1*, and *foxc1*, probably play key roles in gonadal differentiation. Taken together, upregulation of DNA methylation levels of *foxl2* and *cyp19a1a* promoters was associated with gonadal differentiation through regulation of the estradiol-17β synthesis.

## Conclusions

We simultaneously determine both 5mC and 5hmC levels in gonadal genome during gonadal differentiation, and find that overall DNA methylation level and de novo methylase, *dnmt3aa*, are upregulated. We show that transcription factor Foxa1 is responsible for *dnmt3aa* gene expression. In addition, DNA methylation levels of key genes *foxl2* and *cyp19a1a* in regulation of female hormone synthesis are also consistently upregulated during gonadal differentiation. These data pave the way for a better understanding of DNA methylation modification in gonadal differentiation in *Monopterus albus*.

## Supplementary information

**Additional file 1. Table S1.** Primer sequences and PCR conditions. **Table S2.** Linearities of m^5^dC and hm^5^dC by LC-MS analysis.** Table S3.** Mass spectrometry parameters for the analysis of nucleosides.

## Data Availability

Not applicable.

## References

[1] Smallwood SA, Kelsey G (2012). De novo DNA methylation: a germ cell perspective. Trends Genet.

[2] Feng S, Jacobsen SE, Reik W (2010). Epigenetic reprogramming in plant and animal development. Science.

[3] Sasaki H, Matsui Y (2008). Epigenetic events in mammalian germ-cell development: reprogramming and beyond. Nat Rev Genet.

[4] Smallwood SA, Tomizawa S, Krueger F, Ruf N, Carli N, Segonds-Pichon A, Sato S, Hata K, Andrews SR, Kelsey G (2011). Dynamic CpG island methylation landscape in oocytes and preimplantation embryos. Nat Genet.

[5] Gu TP, Guo F, Yang H, Wu HP, Xu GF, Liu W, Xie ZG, Shi L, He X, Jin SG (2011). The role of Tet3 DNA dioxygenase in epigenetic reprogramming by oocytes. Nature.

[6] Tajima S, Suetake I, Takeshita K, Nakagawa A, Kimura H (2016). Domain Structure of the Dnmt1, Dnmt3a, and Dnmt3b DNA Methyltransferases. Adv Exp Med Biol.

[7] Bourc’his D, Xu GL, Lin CS, Bollman B, Bestor TH (2001). Dnmt3L and the establishment of maternal genomic imprints. Science.

[8] Kaneda M, Okano M, Hata K, Sado T, Tsujimoto N, Li E, Sasaki H (2004). Essential role for de novo DNA methyltransferase Dnmt3a in paternal and maternal imprinting. Nature.

[9] Yaman R, Grandjean V (2006). Timing of entry of meiosis depends on a mark generated by DNA methyltransferase 3a in testis. Mol Reprod Dev.

[10] Zamudio N, Barau J, Teissandier A, Walter M, Borsos M, Servant N, Bourc’his D (2015). DNA methylation restrains transposons from adopting a chromatin signature permissive for meiotic recombination. Genes Dev.

[11] Pastor WA, Aravind L, Rao A (2013). TETonic shift: biological roles of TET proteins in DNA demethylation and transcription. Nat Rev Mol Cell Biol.

[12] Wu H, Zhang Y (2014). Reversing DNA methylation: mechanisms, genomics, and biological functions. Cell.

[13] Bochtler M, Kolano A, Xu GL (2017). DNA demethylation pathways: additional players and regulators. BioEssays.

[14] Wu SC, Zhang Y (2010). Active DNA demethylation: many roads lead to Rome. Nat Rev Mol Cell Biol.

[15] Hannenhalli S, Kaestner KH (2009). The evolution of Fox genes and their role in development and disease. Nat Rev Genet.

[16] Jackson BC, Carpenter C, Nebert DW, Vasiliou V (2010). Update of human and mouse forkhead box (FOX) gene families. Hum genomics..

[17] Wijchers PJ, Burbach JP, Smidt MP (2006). In control of biology: of mice, men and Foxes. Biochem J..

[18] Serandour AA, Avner S, Percevault F, Demay F, Bizot M, Lucchetti-Miganeh C, Barloy-Hubler F, Brown M, Lupien M, Metivier R (2011). Epigenetic switch involved in activation of pioneer factor FOXA1-dependent enhancers. Genome Res.

[19] Yang YA, Zhao JC, Fong KW, Kim J, Li S, Song C, Song B, Zheng B, He C, Yu J (2016). FOXA1 potentiates lineage-specific enhancer activation through modulating TET1 expression and function. Nucleic Acids Res.

[20] Cheng H, Guo Y, Yu Q, Zhou R (2003). The rice field eel as a model system for vertebrate sexual development. Cytogenet Genome Res..

[21] Tang Y, Zheng SJ, Qi CB, Feng YQ, Yuan BF (2015). Sensitive and simultaneous determination of 5-methylcytosine and its oxidation products in genomic DNA by chemical derivatization coupled with liquid chromatography-tandem mass spectrometry analysis. Anal Chem.

[22] Xiong J, Ye TT, Ma CJ, Cheng QY, Yuan BF, Feng YQ (2019). N-6-Hydroxymethyladenine: a hydroxylation derivative of N-6-methyladenine in genomic DNA of mammals. Nucleic Acids Rese..

[23] Hong Q, Li C, Ying R, Lin H, Li J, Zhao Y, Cheng H, Zhou R (2018). Loss-of-function of sox3 causes follicle development retardation and reduces fecundity in zebrafish. Protein Cell..

[24] Feng Y, Xie N-B, Tao W-B, Ding J-H, You X-J, Ma C-J, Zhang X, Yi C, Zhou X, Yuan B-F (2020). Transformation of 5-Carboxylcytosine to Cytosine Through C-C Bond Cleavage in Human Cells Constitutes a Novel Pathway for DNA Demethylation. CCS Chemistry..

[25] Hou Y, Yuan J, Zhou X, Fu X, Cheng H, Zhou R (2012). DNA demethylation and USF regulate the meiosis-specific expression of the mouse Miwi. PLoS Genet.

[26] Zhang Y, Zhang S, Liu Z, Zhang L, Zhang W (2013). Epigenetic modifications during sex change repress gonadotropin stimulation of cyp19a1a in a teleost ricefield eel (Monopterus albus). Endocrinology.

[27] Fornes O, Castro-Mondragon JA, Khan A, van der Lee R, Zhang X, Richmond PA, Modi BP, Correard S, Gheorghe M, Baranašić D (2020). JASPAR 2020: update of the open-access database of transcription factor binding profiles. Nucleic Acids Res.

[28] Messeguer X, Escudero R, Farré D, Núñez O, Martínez J, Albà MM (2002). PROMO: detection of known transcription regulatory elements using species-tailored searches. Bioinformatics.

[29] Farré D, Roset R, Huerta M, Adsuara JE, Roselló L, Albà MM, Messeguer X (2003). Identification of patterns in biological sequences at the ALGGEN server: PROMO and MALGEN. Nucleic Acids Res.

[30] Wang DS, Kobayashi T, Zhou LY, Paul-Prasanth B, Ijiri S, Sakai F, Okubo K, Morohashi K, Nagahama Y (2007). Foxl2 up-regulates aromatase gene transcription in a female-specific manner by binding to the promoter as well as interacting with ad4 binding protein/steroidogenic factor 1. Mol Endocrinol.

[31] Rajakumar A, Senthilkumaran B (2020). Steroidogenesis and its regulation in teleost-a review. Fish Physiol Biochem.

[32] Jain D, Meydan C, Lange J, Claeys Bouuaert C, Lailler N, Mason CE, Anderson KV, Keeney S (2017). rahu is a mutant allele of Dnmt3c, encoding a DNA methyltransferase homolog required for meiosis and transposon repression in the mouse male germline. PLoS Genet.

[33] Campos C, Valente LM, Fernandes JM (2012). Molecular evolution of zebrafish dnmt3 genes and thermal plasticity of their expression during embryonic development. Gene.

[34] Zhang Y, Sun X, Zhang L, Zhang W (2017). Testicular Dnmt3 expression and global DNA methylation are down-regulated by gonadotropin releasing hormones in the ricefield eel Monopterus albus. Sci Rep..

[35] Cheng Y, Shang D, Luo M, Huang C, Lai F, Wang X, Xu X, Ying R, Wang L, Zhao Y (2020). Whole genome-wide chromosome fusion and new gene birth in the Monopterus albus genome. Cell Biosci..

[36] Mattiske D, Kume T, Hogan BL (2006). The mouse forkhead gene Foxc1 is required for primordial germ cell migration and antral follicle development. Dev Biol..

[37] Eo J, Han K (2008). K MM, Song H, Lim HJ: Etv5, an ETS transcription factor, is expressed in granulosa and cumulus cells and serves as a transcriptional regulator of the cyclooxygenase-2. J Endocrinol.

