# Supplementary material for: DNA methylation modification is associated with gonadal differentiation in Monopterus albus
Source: Cell Biosci. 2020 Nov 10;10:129. doi: 10.1186/s13578-020-00490-4 (PMC7654577; doi:10.1186/s13578-020-00490-4)
Supplement: Supplementary file 1 — Additional file 1. Table S1. Primer sequences and PCR conditions. Table S2. Linearities of m5dC and hm5dC by LC-MS analysis. Table S3. Mass spectrometry parameters for the analysis of nucleosides. [file 13578_2020_490_MOESM1_ESM.pdf]

Table S1. Primer sequences and PCR conditions

Genes/fragments	Primer sequence (5'-3')	Tm (℃)
dnmt3aa-LS1-F	AATGGTACCACAGAGCAGTACAGACGTACAGA	57
dnmt3aa-LS1-R	AATCTCGAGAAAAAAAGAGACACAAACAGCTA	
dnmt3aa-LS2-F	AATGGTACCAGTGTATCTGCTACACAATTTGT	57
dnmt3aa-LS2-R	AATCTCGAGAAAAAAAGAGACACAAACAGCTA	
dnmt3aa-LS3-F	AATGGTACCAATTTATTCAAAAAAGACAAATCTA	57
dnmt3aa-LS3-R	AATCTCGAGAAAAAAAGAGACACAAACAGCTA	
dnmt3aa-LS4-F	AATGGTACCCATTTGGACATTTTGTGTCCTTT	57
dnmt3aa-LS4-R	AATCTCGAGAAAAAAAGAGACACAAACAGCTA	
Mut a-F	GCAGGTCTCTTTTTTTCTCGAGATCTGCG	62
Mut a-R	GAGTCTACAAAGAGATGGAAAAATGACCA	
Mut b-F	CAGCGACTCGCAGGTCTCTTTTTTTCTC	62
Mut b-R	CGTGGATGGAAAAATGACCATAAAAAGA	
foxa1-CDS-F	AATGAATTCATGCTGGGCACGGTGAAGA	58
foxa1-CDS-R	AATCTCGAGCTATGAGGTGTTGAGGAGCGGT	
foxa1-qPCR-F	ATGTTTGAGAACGGCTGCTAC	58
foxa1-qPCR-R	AGTAATGGGGGTCTCCTTTGA	
dnmt3aa-qPCR-F	CTGCTGTAGGTGTTTCTGTGT	60
dnmt3aa-qPCR-R	AAATCCTTCAGCACCAACAGT	
Chip-P1-F:	AATGGTACCCATTTGGACATTTTGTGTCCTTT	57
Chip-P1-R:	CAAAGTGACTACAAAGAAACC	
Chip-P2-F:	GAGATGGCGACCTCTGACC	57
Chip-P2-R:	CCTTGTTCCTCTCAGTTTG	
hprt-qPCR-F	GAACAGTGACCGCTCCATCC	58
hprt-qPCR-R	TTGTCAGGGACCTCGAATCCT	
foxl2-qPCR-F	CTCAGTCTCAACGAATGCTTC	57
foxl2-qPCR-R	CACGTTCACTGGACTTACGTT	
cyp19a1a-qPCR-F	GCATGAACGAGAGAGGCATTA	57
cyp19a1a-qPCR-R	TAGATGTCCGGTTTAAGCAGC	

foxl2-BSP-F	TTAATTTAAAAAGTATAATAGAAGGAAA	52
foxl2-BSP-R	TTTCAAATCACTAACAAACTACCTT	
foxl2-BSP-Rn	TTTACTCCAATACTATCCATAAA	

Table S2. Linearities of m^5dC and hm^5dC by LC-MS analysis.

Analytes	Linear range (analyte/dC %)	Calibration curve		R^2
		Slope	Intercept	
m^5dC	0.2-10	0.0436	0.0103	0.9954
hm^5dC	0.01-0.5	0.0574	8×10^{-6}	0.9967

Table S3. Mass spectrometry parameters for the analysis of nucleosides.

Analytes	Precursor ion	Product ion	DP/V	EP / V	CE / V	CXP / V
dC	228.2	112.1	22.0	4.0	15.0	4.0
m^5dC	242.2	126.1	22.0	4.5	21.0	3.0
hm^5dC	258.2	142.2	20.0	4.0	15.0	3.0
	258.2	124.2	20.0	5.0	30.0	4.0
